# Supplementary figures and images for: A systems biology approach uncovers the core gene regulatory network governing iridophore fate choice from the neural crest
Source: PLoS Genet. 2018 Oct 4;14(10):e1007402. doi: 10.1371/journal.pgen.1007402 (PMC6191144; doi:10.1371/journal.pgen.1007402)

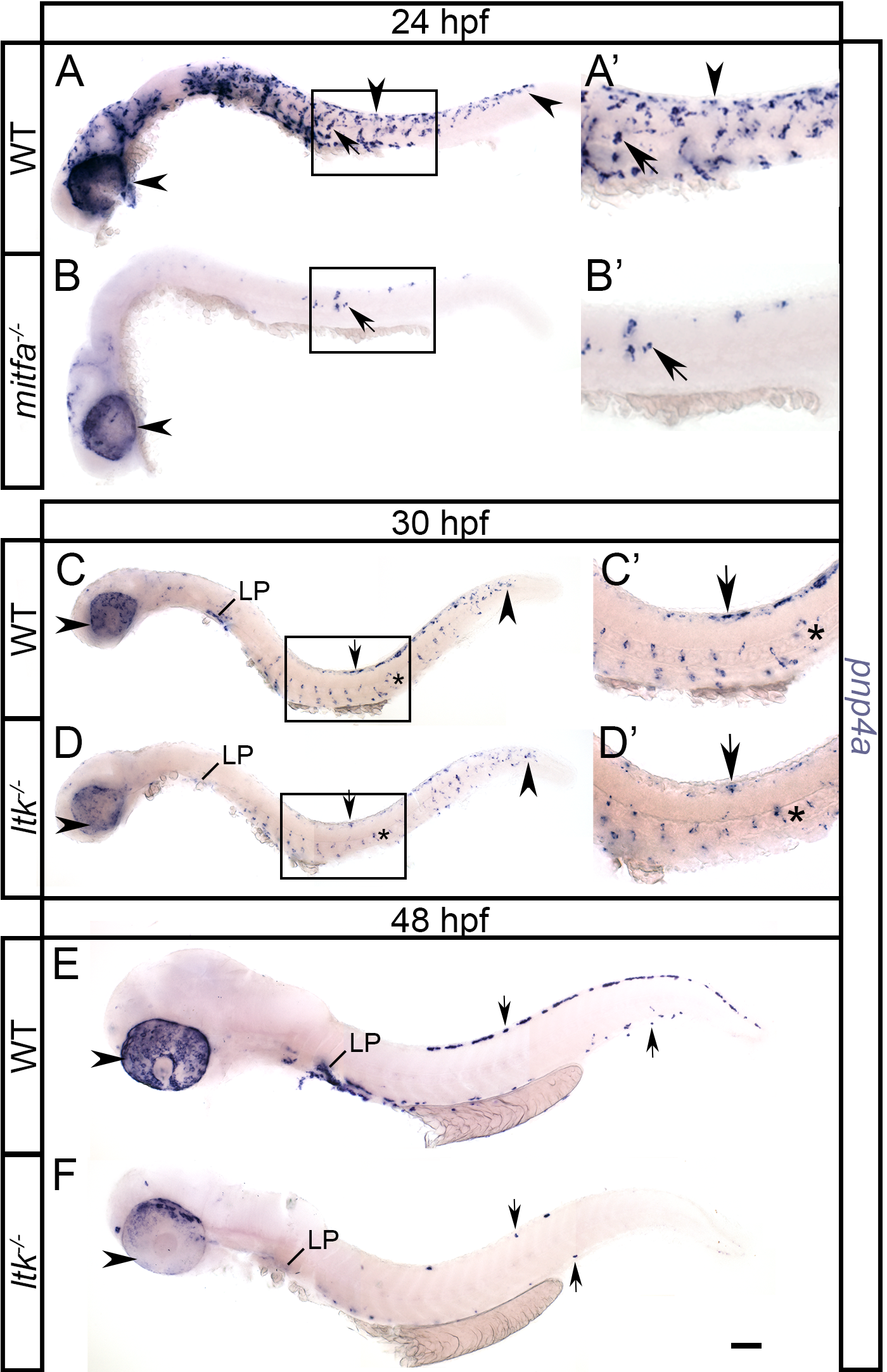

Supplement: S1 Fig — Chromogenic WISH at 24 hpf shows almost complete elimination of pnp4a expression from the NC derivatives of the dorsal trunk (vertical arrowheads) and the migratory pathways (arrows) of mitfa mutants (B, B’), compared to WT siblings (A, A’). Expression in the RPE domain is reduced (horizontal arrowheads). WISH at 30 hpf (C-D’) reveals persistence of pnp4a expression in migrating cells which we interpret as melanoblasts, in ltk mutants (asterisks), as well as in the multipotent progenitor domain of the posterior tail (vertical arrowheads). We also observe a reduced number of cells in iridoblast locations: overlying the RPE (horizontal arrowheads), in the developing lateral patches and along the dorsal posterior trunk (arrows, enlarged in C’,D’). At 48 hpf (E,F), the majority of pnp4a-positive cells in iridophore locations are absent in ltk mutants. Specifically, cells overlying the RPE (horizontal arrowheads), on the lateral patches and along the dorsal and ventral posterior trunk and tail (arrows) are dramatically reduced. Very few escaper iridophores (F, arrows) maintain strong pnp4a expression upon loss of ltk function. LP, lateral patches. Lateral views, heads positioned towards the left. Scale bar corresponds to 100 μm in A,B,C,D,E,F and to 50 μm in A’,B’,C’,D’. (TIF) [file pgen.1007402.s001.tif]

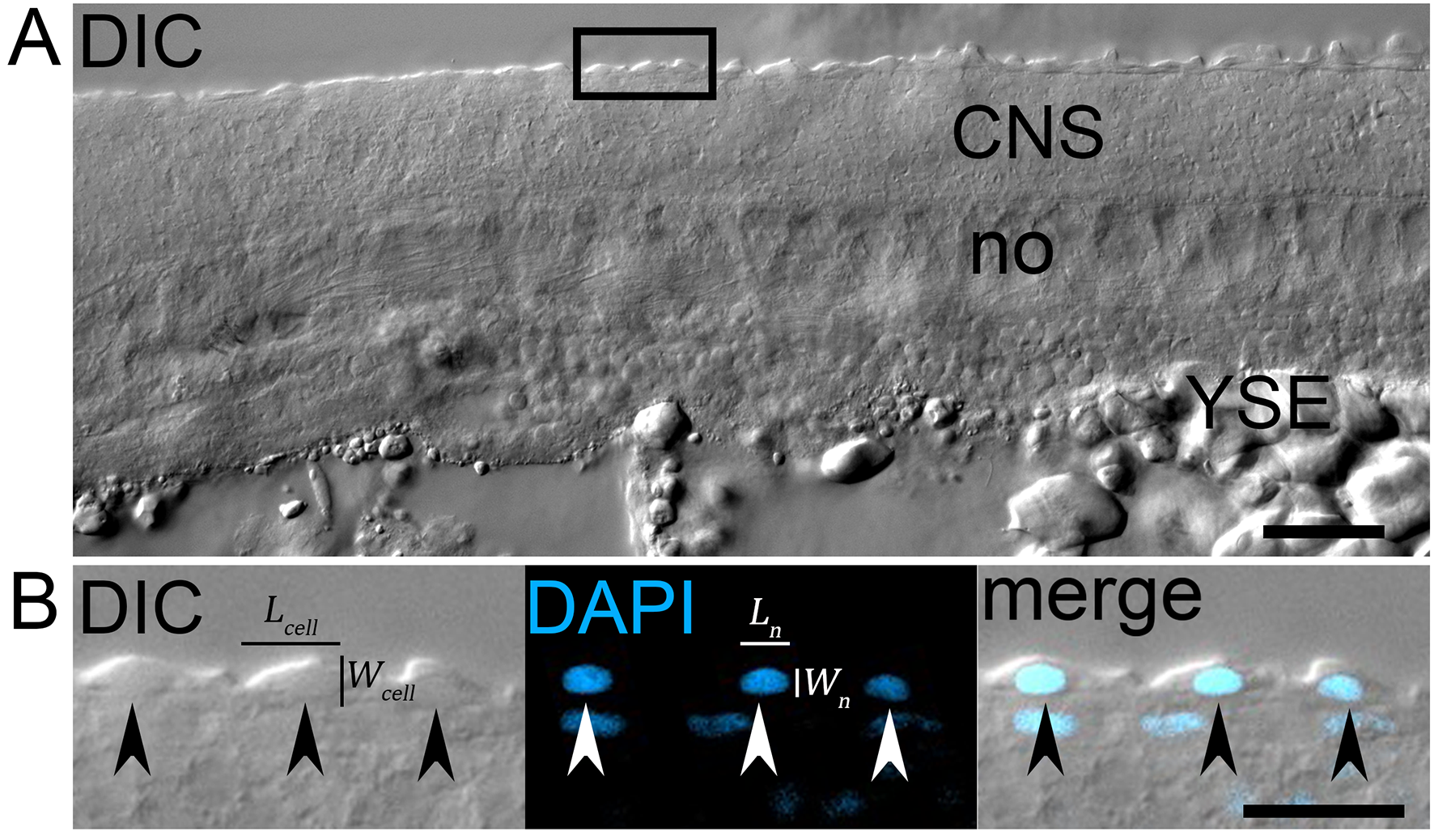

Supplement: S2 Fig — (A) DIC image of a single focal plane from a Z-stack, showing the ARPT of a 24 hpf WT embryo. (B) Magnified view of the boxed region in (A). DIC allows for identification of the boundaries of the cells directly dorsal to the CNS (likely epidermal), while DAPI stain renders the nuclei visible. It is thus possible to measure the length (L) and width (W) of whole cells (arrowheads), as well as of their respective nuclei. CNS, central nervous system; no, notochord; YSE, yolk sac extension. Lateral view, head positioned towards the left. Scale bar: A: 50 μm; B: 20 μm. (TIF) [file pgen.1007402.s002.tif]

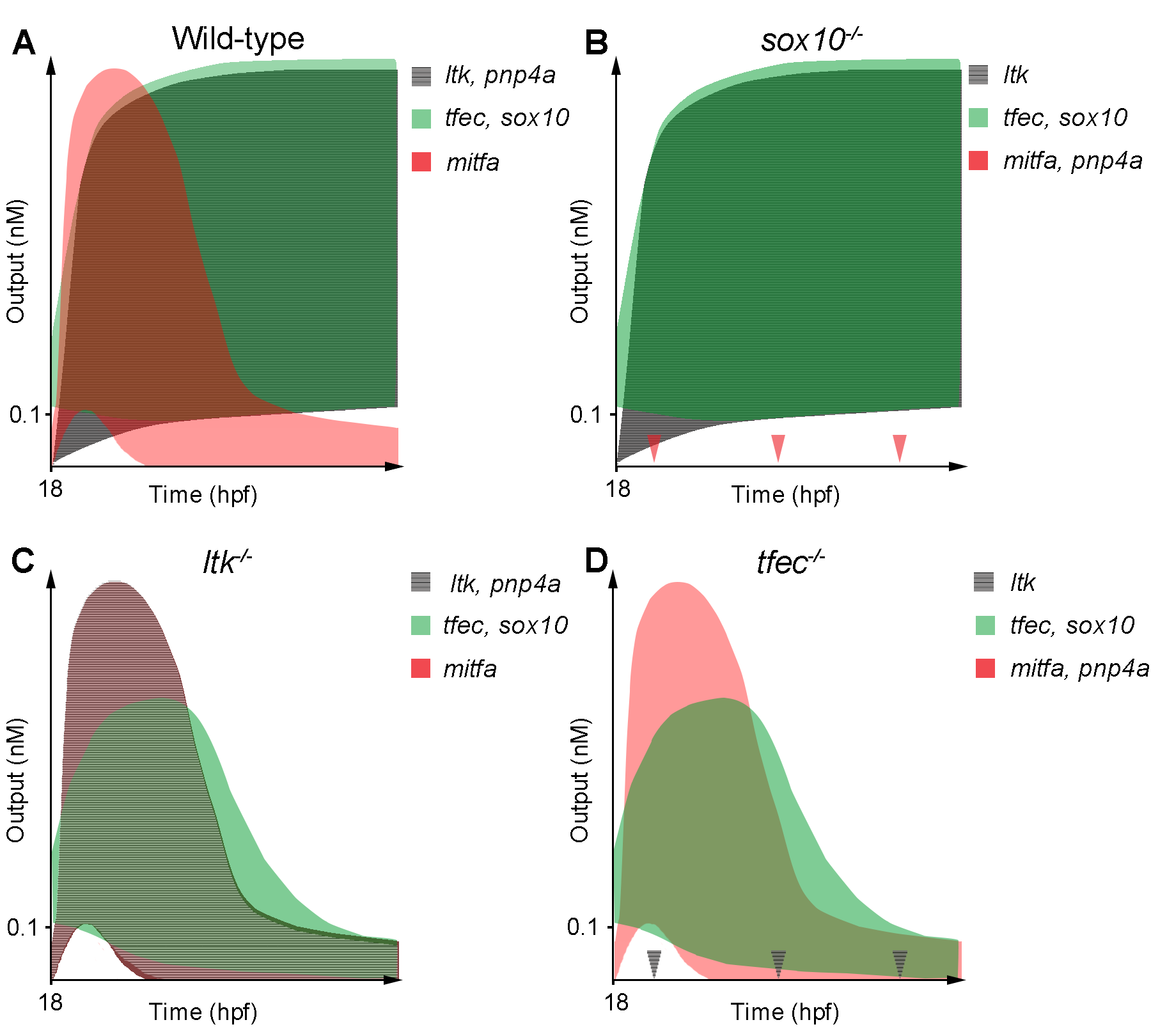

Supplement: S3 Fig — Our experimental data suggest that (A) in WT embryos, Ltk and Pnp4a concentrations rise from undetectable initial levels ([L], [P] = 0 at 18 hpf) to an unknown steady state (grey region). We arbitrarily set the threshold of detectability at 0.1 nM. Mitfa initially rises to levels that are unknown, but above the detection threshold, then plateaus at a level below detection (red region). Tfec and Sox10 concentrations are unknown, yet above the detection threshold, at 18 hpf. We expect them to either remain constant or rise to a higher plateau during iridophore development (green region). (B) In sox10 loss of function mutants, we expect Tfec (green region) and Ltk (grey region) concentrations to remain detectable in the trapped iridoblast progenitors, but for both Pnp4a and Mitfa to not become upregulated (red arrowheads). (C) ltk mutant simulations are expected to show a WT-like rise and fall of Mitfa concentration (red region); Ltk and Pnp4a will also rise from initially undetectable, to levels above the detection threshold, followed by gradual reduction below that threshold (grey region). Tfec and Sox10, initially present in the progenitors, are both expected to gradually decline and become undetectable at later stages of iridophore development (green region). (D) In the tfec loss of function context, we expect Mitfa and Pnp4a to rise from undetectable to levels above detection threshold, then subsequently decline below detection threshold (red region); Tfec and Sox10 also decline as iridoblasts fail to develop beyond the initial specification phase (green region). Ltk is never upregulated in this mutant (grey arrowheads). (TIF) [file pgen.1007402.s003.tif]

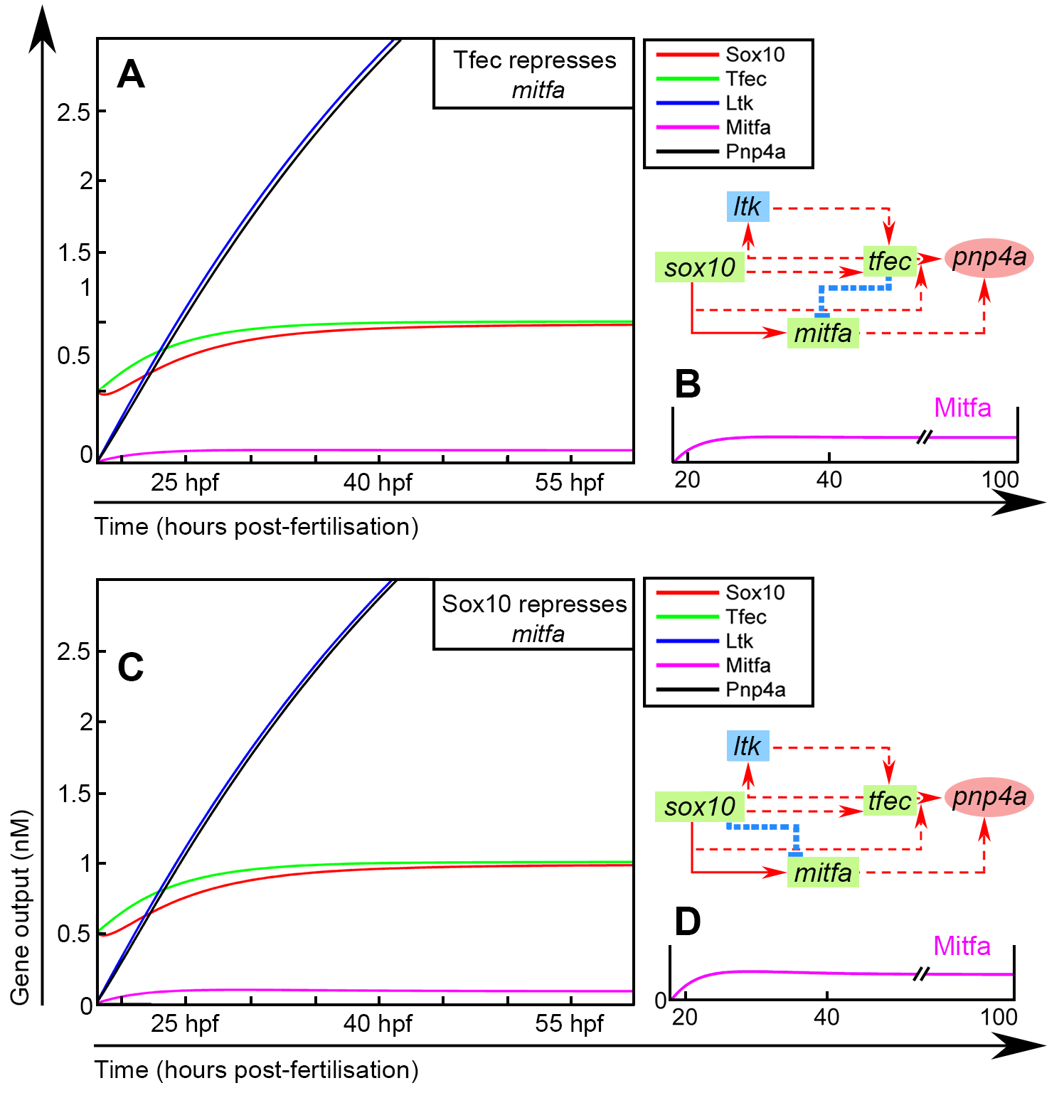

Supplement: S4 Fig — Testing alternative methods of repressing mitfa expression using the mathematical model failed to recapitulate the experimentally observed mitfa dynamics in the absence of factor R. (A,B) Implementing Tfec-dependent suppression of mitfa resulted only in a relatively lower positive plateau of Mitfa output, instead of a peak at approximately 24 hpf, followed by downregulation of mitfa. (C,D) Implementing Sox10-dependent suppression of mitfa resulted in the same outcome. (TIF) [file pgen.1007402.s004.tif]

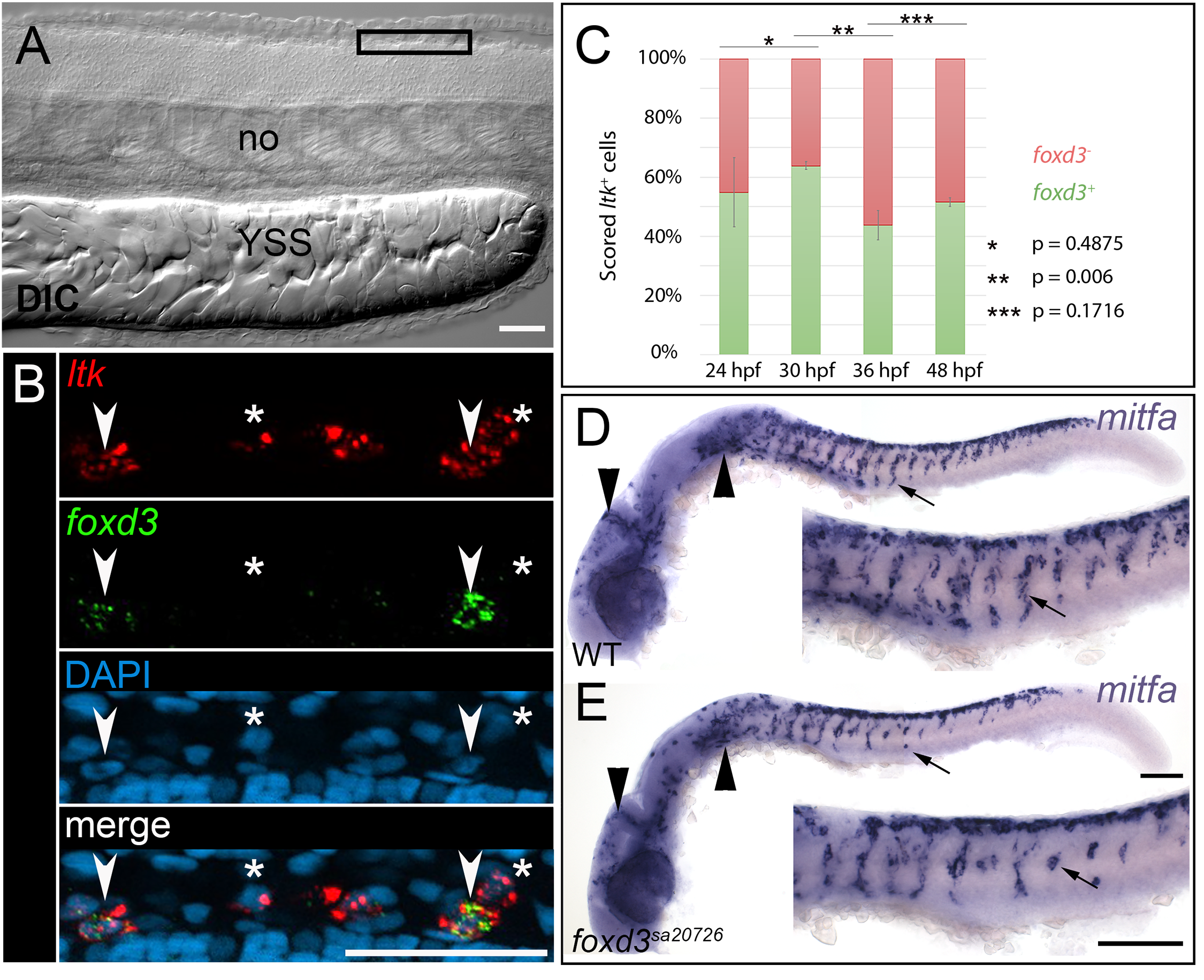

Supplement: S5 Fig — (A, B) RNAscope experiments at 36 hpf reveal that foxd3 does not fulfil the criteria established using our models for factor R, as it is only expressed in a subset of ltk+ ib(df) of the posterior dorsal trunk. In (B) arrowheads point at ltk+ cells that co-express foxd3, while asterisks indicate cells that are only positive for ltk. (C) RNAscope experiments at 24 hpf, 30 hpf, 36 hpf and 48 hpf indicate that 55%, 64%, 44% and 52%, respectively, of ltk+ cells co-express foxd3. t-tests suggest that the proportion of cells co-expressing the two genes only significantly changed between 30 hpf and 36 hpf. (D,E) foxd3 mutants subjected to WISH at 24 hpf present with a reduction of mitfa+ cells along the migratory pathways of the posterior trunk. WT and mutant embryos show no difference in cranial NC derived mitfa+ populations (arrowheads), but noticeable and consistent decrease of migrating trunk NC derivatives (*arrows). no, notochord; YSS, yolk sac stripe. Lateral views, head towards the left. Scale bars: (A,B) 50 μm, (D,E) 100 μm. (TIF) [file pgen.1007402.s005.tif]

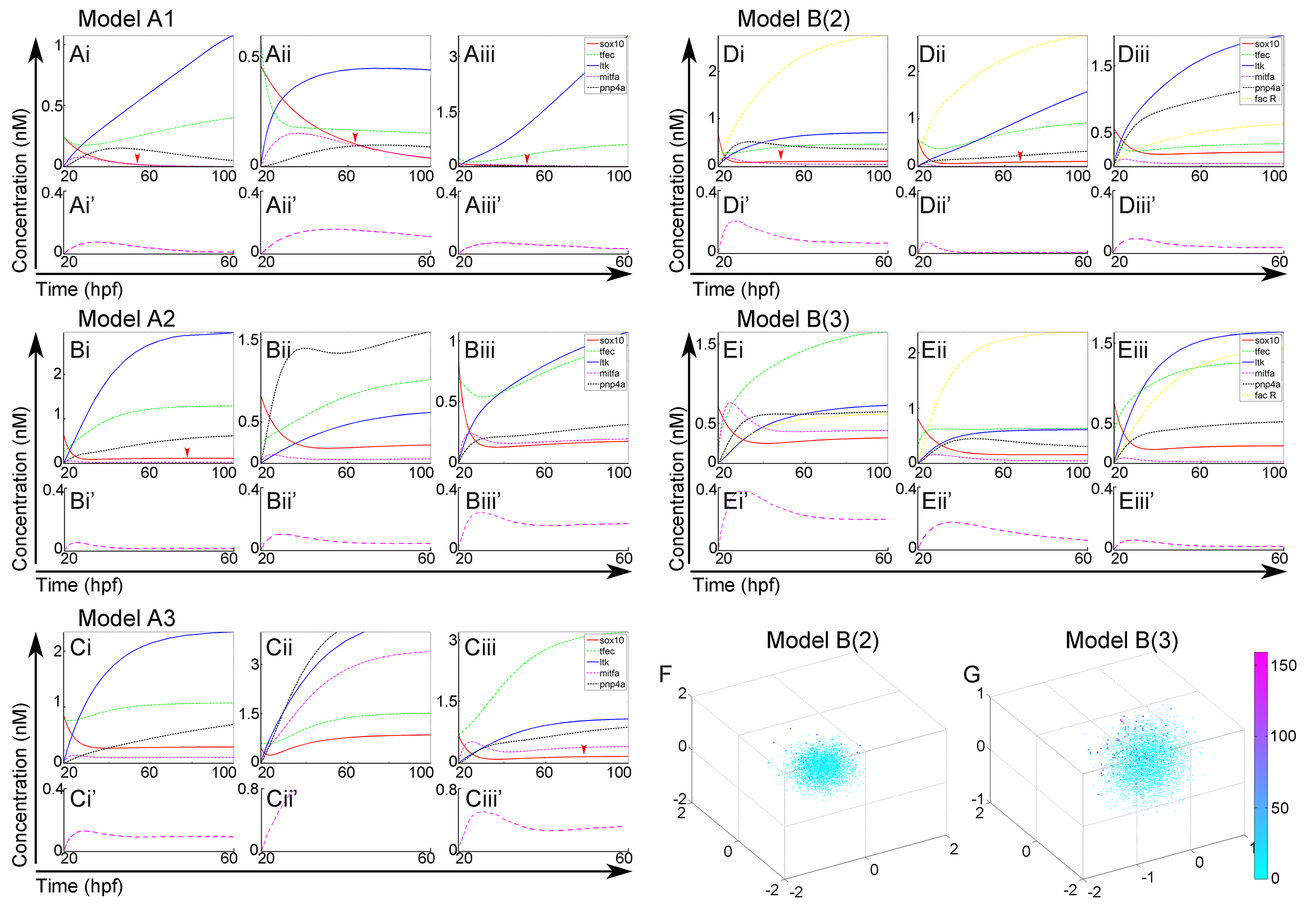

Supplement: S6 Fig — WT outputs derived from the three best scoring parameter combinations (i, ii, iii) following 20,000 runs using Monte Carlo for models A1 (A), A2 (B), A3 (C), B(2) (D) and B(3) (E; referred to as model B in this work). (i’, ii’ and iii’) represent magnified mitfa expression dynamics in each of the outputs. In model A1 (Ai-Aiii’), a very subtle rise and drop of Mitfa concentration is only achievable if Sox10 concentration reaches undetectable levels at steady-state (red arrowheads). In (Bi) Mitfa remains very low throughout iridoblast specification ([M]<0.1 nM), while in (Bii), (Biii) the decline is very subtle and mitfa remains upregulated in steady state. (Ci) and (Ciii) show Mitfa staying relatively high at steady state, compared to the achieved maximum levels, with Sox10 declining below detection level in (Ciii). In (Cii) Mitfa is steadily upregulated to its steady-state concentration value. In the two highest scoring model B(2) outputs (where factor R was incorporated in model A2), Mitfa exhibits the required rise and drop dynamics only when Sox10 declines (Di, Dii; red arrowheads). The WT outputs of the three top scoring trials for model B(3) (derived from A3), exhibit maintenance of sox10 expression, while Mitfa peaks at approximately 24 hpf, before declining to no more than half the maximum value (Ei-Eiii’). PCA analysis of all Monte Carlo outputs for this model (F) reveals that significantly fewer trials score high (dark blue and magenta spots) and that the absolute score value is lower, compared to trials using model B(3), i.e. the chosen model B (G). (TIF) [file pgen.1007402.s006.tif]
