# Supplementary material for: A systems biology approach uncovers the core gene regulatory network governing iridophore fate choice from the neural crest
Source: PLoS Genet. 2018 Oct 4;14(10):e1007402. doi: 10.1371/journal.pgen.1007402 (PMC6191144; doi:10.1371/journal.pgen.1007402)
Supplement: S1 Table — The Pearson’s chi-squared test for goodness of fit indicates the likelihood that embryos presenting with an expression pattern differing from the WT might correspond to homozygous mutants of the respective allele (indicated in the second column). All the alleles follow the classic Mendelian ratios, thus 25% of the total of examined embryos are expected to be homozygous mutants. For each of the four stages, the 1st sub-column presents the number of embryos out of the examined total which show a characteristic alternative expression pattern, and the 2nd indicates the associated p-value. If p > 0.1 we accept the null hypothesis that any deviation in the observed number of embryos with an alternative phenotype from the expected number of homozygous mutants in the sample is only due to random chance. In red/bold are any samples where observed phenotypes did not significantly correlate with expected Mendelian ratios, i.e. where there is unlikely to be a mutant phenotype. (PDF) [file pgen.1007402.s007.pdf]

WISH pattern	Genotype	Stages							
		18 hpf		24 hpf		30 hpf		48 hpf	
tfec	*sox10*$^{-/-}$	**0 of 41**	**$p < 0.001$**	20 of 74	$0.6 < p < 0.7$	17 of 66	$p \approx 0.9$	12 of 59	$0.3 < p < 0.5$
	ltk$^{-/-}$	**0 of 79**	**$p < 0.001$**	**5 of 194**	**$p < 0.001$**	23 of 80	$0.3 < p < 0.5$	29 of 99	$p \approx 0.7$
	tfec$^{-/-}$			14 of 48	$p \approx 0.5$	15 of 51	$0.3 < p < 0.5$	9 of 38	$0.8 < p < 0.9$
ltk	*tfec*$^{-/-}$			12 of 49	$p \approx 0.95$	16 of 46	$0.1 < p < 0.2$	13 of 36	$0.1 < p < 0.2$
pnp4a	*sox10*$^{-/-}$			13 of 45	$0.5 < p < 0.7$	8 of 39	$0.4 < p < 0.5$	11 of 33	$0.2 < p < 0.3$
	ltk$^{-/-}$			**6 of 105**	**$p < 0.001$**	9 of 42	$0.5 < p < 0.7$	7 of 28	$p > 0.95$
	tfec$^{-/-}$			11 of 49	$p \approx 0.7$	10 of 41	$p \approx 0.95$	9 of 44	$p \approx 0.5$
	mitfa$^{-/-}$			11 of 49	$0.5 < p < 0.7$	11 of 31	$0.1 < p < 0.2$		
mitfa	*foxd3*$^{-/-}$			12 of 56	$0.5 < p < 0.7$				

S1 Table. Statistics of loss of function experiments.
