# Supplementary material for: A systems biology approach uncovers the core gene regulatory network governing iridophore fate choice from the neural crest
Source: PLoS Genet. 2018 Oct 4;14(10):e1007402. doi: 10.1371/journal.pgen.1007402 (PMC6191144; doi:10.1371/journal.pgen.1007402)
Supplement: S2 Table — The default parameter set, selected as physiologically relevant based on published literature. These parameters were used for models A1, A2, A3 and B. For references see S1 Text. (PDF) [file pgen.1007402.s008.pdf]

	K_d (nM)	g (nM/h)	d (1/h)
sox10/ Sox10	0.1	0.2	0.2
tfec/ Tfec	0.1	0.2	0.2
mitfa/ Mitfa	0.1	0.2	0.2
ltk/ Ltk	0.1	0.2	0.03
pnp4a/ Pnp4a		0.2	0.03
factor R/ Factor R	0.1	0.2	0.2

S2 Table. Parameter Choice.
