# Supplementary material for: A systems biology approach uncovers the core gene regulatory network governing iridophore fate choice from the neural crest
Source: PLoS Genet. 2018 Oct 4;14(10):e1007402. doi: 10.1371/journal.pgen.1007402 (PMC6191144; doi:10.1371/journal.pgen.1007402)
Supplement: S3 Table — Presence of foxd3 transcripts in ltk+ iridophore lineage cells was assessed along the trunks of embryos at 24, 30, 36 and 48 hpf using RNAscope. For each stage, the total number of wild-type embryos used for scoring and the total number of ltk+ cells scored for foxd3 expression are recorded. The percentage of these cells expressing even very low levels (>2 spots of fluorescence surrounding the nucleus) of foxd3 is shown, along with the associated standard error of the mean, calculated via the standard deviation from each experimental replicate. * Note that for the 48 hpf experiment, the SEM column contains the standard deviation value. (PDF) [file pgen.1007402.s009.pdf]

Markers analysed	Stage (hpf)	No. of scored embryos	No. of scored *ltk*+ (L) or *tfec*+ (T) cells	Mean % of co-expressing cells	SEM (*)/ SD (**)
foxd3; ltk	24	5	128 (L)	54.9%	11.65 (*)
	30	4	183 (L)	63.8%	1.26 (*)
	36	7	216 (L)	43.8%	4.89 (*)
	48	3	126 (L)	51.6%	2.75 (*)
tfec; ltk	24	2	41 (T)	88.4%	8.39 (**)
	30	2	95 (T)	99.0%	1.00 (**)
	48	1	50 (T)	100.0%	
tfec; mitfa	24	3	42 (T)	89.7%	1.71 (**)
	30	3	52 (T)	23.1%	10.18 (**)
	48	3	47 (T)	16.6%	5.34 (**)
ltk; sox10	**24**	2	111 (L)	86.8%	3.55 (**)
	30	2	102 (L)	89.6%	5.15 (**)
	48	3	112 (L)	91.3%	3.96 (**)

S3 Table. Quantification of cells co-expressing different markers by RNAscope.
