# Supplementary material for: A systems biology approach uncovers the core gene regulatory network governing iridophore fate choice from the neural crest
Source: PLoS Genet. 2018 Oct 4;14(10):e1007402. doi: 10.1371/journal.pgen.1007402 (PMC6191144; doi:10.1371/journal.pgen.1007402)
Supplement: S1 Text — (PDF) [file pgen.1007402.s010.pdf]

Description of biological variables:

In accordance to [1], we describe constant parameters and time-dependent variables as follows.

The rate at which the output of each gene (G) is produced is derived as the product of the maximum mRNA production rate (g_G, measured in nM/h) and the average number of active copies of gene G across a homogeneous population of cells ($[G^*]$). The parameter constant g_G further incorporates the rates of post-transcriptional modifications, protein synthesis and post-translational modifications, which are not being modelled explicitly here. At any given time, we consider the homogeneous population of cells experimentally assigned to the iridophore lineage, during a particular developmental stage, based on their molecular signatures, position along the axis of the embryo and morphology (Fig. 1; Fig. 7). The dynamics of $[G^*]$ incorporates all individual activatory and repressive inputs acting on gene G and is calculated according to the following:

- If gene G is only activated by transcription factor A, then the active genes are those whose regulatory elements have been occupied by factor A, thus:

$$[G^*] = \left[r_A^G\right] \tag{1}$$

Where $\left[r_A^G\right]$ indicates the average number of regulatory elements of gene G that are occupied by transcription factor A in the homogeneous population of cells. This is calculated using the following equation, according to Greenhill et al. [1]:

$$\left[r_A^G\right] = \frac{[A]}{K_A^G + [A]} \tag{2}$$

In equation (2) $[A]$ indicates the concentration of gene A protein (measured in nM) and K_A^G is the dissociation constant describing the binding affinity of transcription factor A for the promoter of gene G. As this model does not distinguish between direct and indirect interactions, this parameter is used to indicate the strength of activation of gene G by factor A.

- If transcription factors A and B are independently capable of activating gene G by binding to distinct regulatory elements (non-competitive activation), then $[G^*]$ equals the probability $p(G^*)$ that gene G has become activated either by a single or by both factors. $p(G^*)$ is measured as the sum of the following individual probabilities:

  (a) that only transcription factor A has bound to gene G elements $p(A, \sim B)$,

  (b) that only transcription factor B has bound to gene G elements $p(\sim A, B)$, and

  (c) that factors A and B have simultaneously bound to gene G elements $p(A, B)$.

Thus:

$$\begin{aligned} [G^*] = p(G^*) &= p(A, \sim B) + p(\sim A, B) + p(A, B) = \\ &= \left[r_A^G\right]\left(1 - \left[r_B^G\right]\right) + \left[r_B^G\right]\left(1 - \left[r_A^G\right]\right) + \left[r_A^G\right]\left[r_B^G\right] = \\ &= \left[r_A^G\right] + \left[r_B^G\right]\left(1 - \left[r_A^G\right]\right) \end{aligned} \tag{3}$$

- If binding of both transcription factors A and B is required to activate gene G, then the probability that gene G is in the active state equals the product of the probabilities of each transcription factor binding:

$$[G^*] = p([G^*]) = p(A,B) = [r_A^G]\,[r_B^G] \tag{4}$$

- If transcription factor A activates and factor B represses gene G, then the probability that gene G is in the active state equals the probability that factor A is bound and factor B not bound at the same time:

$$[G^*] = p([G^*]) = p(A,\sim B) = [r_A^G]\,(1 - [r_B^G]) \tag{5}$$

For more details on the derivation of the above, refer to the supplementary information of [1]. Finally, the rate of degradation of the protein product of gene G is calculated according to the Law of Mass Action as the product of the mean concentration of protein in the cell at a given time across the homogeneous cell population ($[G]$, measured in nM) and a parameter constant d_G indicating the rate of degradation of $[G]$, measured in 1/h.

Estimation of parameter values

The constant parameters g_G, d_G and K_A^G were assigned physiologically relevant values according to previously published literature.

Maximum mRNA production rate

Global gene expression analysis [2] estimated the median of cellular transcription rates to be 2 mRNA molecules per hour. To derive the maximum gene G mRNA production rate (g_G, nM/h), we first calculated the average cytoplasmic volume (V_{cyt}) of cells positioned in the beginning of the posterior dorsal trunk at each of 18 hpf, 24 hpf, 36 hpf and 4 dpf (summarised in table ST1). To achieve this, we measured the width (W_{cell}) and length (L_{cell}) of each cell, as well as the width (W_n) and length (L_n) of the respective DAPI-stained nuclei, using the FIJI software. Based on DIC Z-stack visualisation (S2 Fig.), we assumed that cells are shaped roughly as rods with rounded ends, thus the equation used to calculate the cytoplasmic volume was as follows:

$$V_{cyt} = \pi\left(W_{cell}{}^2\,\frac{L_{cell} - (W_{cell}/3)}{4} - W_n{}^2\,\frac{L_n - (W_n/3)}{4}\right) \tag{6}$$

ST1 Table. Calculation of mean cytoplasmic volume in cells positioned in NC and iridophore lineage positions at different developmental stages. The final column indicates the average value across all developmental stages.

Stage (hpf)	18	24	36	4	
No. of cells scored	5	8	7	6	26 (total)
Mean V_{cyt} (μm^3)	186.97	257.41	126.19	73.88	161.11 (average)

Across stages, the average cytoplasmic volume was 161.11 μm^3, or 0.16 pL.

$$1\,molecule = 1.7 \times 10^{-15} nmoles \tag{7}$$

Hence,

$$2\,\frac{molecules}{hour} = 3.4 \times 10^{-15}\,\frac{nmoles}{hour} \tag{8}$$

By dividing with the average cytoplasmic volume ($V_{cyt} = 0.16 \times 10^{-12} L$) we obtain that the median rate of 2 molecules per hour [2] corresponds to 0.02 nM/hour. We arbitrarily assumed the maximum

gene expression rate to be 10 times higher than the calculated median rate. Thus, for all genes the following maximum production rate was set to

$$g_G = 0.2 \frac{nM}{h} \tag{9}$$

Protein degradation rate

Protein half-lives (τ) have been experimentally identified by others [2]–[5]. We converted the identified half-lives to degradation rates according to published instructions [6] using the equation

$$d_G = \frac{\ln(2)}{\tau} \tag{10}$$

Transcription factors have been found to degrade faster on average compared to other proteins [4]. Therefore, we used the estimated half-life of human SOX9 ($\tau = 3.6\ h$; Mertin, McDowall, and Harley 1999) to derive a physiologically relevant degradation rate for all transcription factors in the model (d_{TF}). Using (10), this was $d_{TF} = 0.2\ \ 1/h$.

For transmembrane receptors and enzymes we used the highest half-life value which was consistently reported ($\tau = 22.5\ h$;) [2], [5]. Using (10), the resulting degradation rate was $d_{R,E} = 0.03\ \ 1/h$.

Dissociation constant

We assumed the dissociation constant K for all genes to be $K = 0.1\ nM$. This choice is within the physiologically relevant range according to published literature [3], [7]–[9]. The chosen value allowed for gene outputs to reach positive stable steady states in WT simulations, as expected in the iridophore lineage. Using values higher than 0.1 nM (corresponding to lower binding affinities) prevented successful upregulation of genes (data not shown).

Model A1

sox10 is activated by Sox10

The rate of change of Sox10 output depends upon direct or indirect activation by Sox10 itself. By using $[S]$ to indicate the concentration of Sox10, this translates into the equation:

$$\frac{d[S]}{dt} = g_S\,[S^*] - \, d_S\,[S] \tag{11}$$

Because of (1), we obtain (16).

mitfa is activated by Sox10

The rate of change of Mitfa output depends upon direct activation of *mitfa* by Sox10. By using $[M]$ to indicate the concentration of Mitfa, this translates into the equation:

$$\frac{d[M]}{dt} = g_M\,[M^*] - d_M\,[M] \tag{12}$$

Because of (1), we obtain (17).

tfec is activated by Sox10 and Ltk

The rate of change of Tfec output depends upon direct or indirect activation of *tfec* by Sox10 or upon indirect activation by Ltk. By using $[T]$ to indicate the concentration of Tfec, this translates into the equation:

$$\frac{d[T]}{dt} = g_T\,[T^*] - \, d_T\,[T] \tag{13}$$

Because of (3), we obtain (18).

ltk is activated by Tfec

The rate of change of Ltk output depends upon direct or indirect activation of *ltk* by Tfec. By using $[L]$ to indicate the concentration of Ltk, this translates into the equation:

$$\frac{d[L]}{dt} = g_L\,[L^*] - \, d_L\,[L] \tag{14}$$

Because of (1), we obtain (19).

pnp4a is activated by Sox10 AND Tfec and by Mitfa

The rate of change of Pnp4a output depends upon direct or indirect activation by simultaneous action of Tfec and Sox10, or upon direct or indirect activation by Mitfa. By using $[P]$ to indicate the concentration of Pnp4a, this translates into the equation:

$$\frac{d[P]}{dt} = g_P\,[P^*] - \, d_P\,[P] \tag{15}$$

Because of (3) and (4), we obtain (20).

Model A1 system of ODEs

$$\begin{cases} \frac{d[S]}{dt} = g_S\,[r_S^S] - d_S\,[S] & (16) \\ \frac{d[M]}{dt} = g_M\,[r_S^M] - d_M\,[M] & (17) \\ \frac{d[T]}{dt} = g_T\,([r_S^T](1 - [r_L^T]) + [r_L^T]) - d_T\,[T] & (18) \\ \frac{d[L]}{dt} = g_L\,[r_T^L] - d_L\,[L] & (19) \\ \frac{d[P]}{dt} = g_P\,([r_S^P]\,[r_T^P] + [r_M^P] - [r_S^P]\,[r_T^P]\,[r_M^P]) - d_P\,[P] & (20) \end{cases}$$

Model A2

sox10 is activated by Tfec

The rate of change of Sox10 output depends upon direct or indirect activation by Tfec.

From equations (11) and (1), we obtain (21).

Model A2 system of ODEs

$$\begin{cases} \dfrac{d[S]}{dt} = g_S\,[r_T^S] - d_S\,[S] & (21) \\ \dfrac{d[M]}{dt} = g_M\,[r_S^M] - d_M\,[M] & (22) \\ \dfrac{d[T]}{dt} = g_T\,([r_S^T](1 - [r_L^T]) + [r_L^T]) - d_T\,[T] & (23) \\ \dfrac{d[L]}{dt} = g_L\,[r_T^L] - d_L\,[L] & (24) \\ \dfrac{d[P]}{dt} = g_P\,([r_S^P]\,[r_T^P] + [r_M^P] - [r_S^P]\,[r_T^P]\,[r_M^P]) - d_P\,[P] & (25) \end{cases}$$

Model A3

sox10 is activated by Ltk

The rate of change of Sox10 output depends upon direct or indirect activation by Sox10 itself.

From equations (11) and (1), we obtain (26).

Model A3 system of ODEs

$$
\begin{cases}
\dfrac{d[S]}{dt} = g_S\,[r_L^S] - d_S\,[S] & (26) \\[2ex]
\dfrac{d[M]}{dt} = g_M\,[r_S^M] - d_M\,[M] & (27) \\[2ex]
\dfrac{d[T]}{dt} = g_T\,([r_S^T](1-[r_L^T]) + [r_L^T]) - d_T\,[T] & (28) \\[2ex]
\dfrac{d[L]}{dt} = g_L\,[r_T^L] - d_L\,[L] & (29) \\[2ex]
\dfrac{d[P]}{dt} = g_P\,([r_S^P]\,[r_T^P] + [r_M^P] - [r_S^P]\,[r_T^P]\,[r_M^P]) - d_P\,[P] & (30)
\end{cases}
$$

Model B

Factor R is activated by Tfec

The rate of change of Factor R output depends upon its direct or indirect activation by Tfec. By using $[FR]$ to indicate the concentration of Factor R, this translates into the equation:

$$\frac{d[FR]}{dt} = g_{FR}\,[FR^*] - d_{FR}\,[FR] \tag{31}$$

Because of (1), we obtain (32).

mitfa is activated by Sox10 and repressed by factor R

The rate of change of Mitfa output depends upon direct activation of *mitfa* by Sox10, combined with repression by factor R.

From equations (12) and (5), we obtain (33).

Model B system of ODEs

$$\left\{\begin{aligned}
\frac{d[S]}{dt} &= g_S\,[r_L^S] - d_S\,[S] && (32)\\
\frac{d[M]}{dt} &= g_M\left([r_S^M]\,(1 - [r_{FR}^M])\right) - d_M\,[M] && (33)\\
\frac{d[T]}{dt} &= g_T\,([r_S^T]\,(1 - [r_L^T]) + [r_L^T]) - d_T\,[T] && (34)\\
\frac{d[L]}{dt} &= g_L\,[r_T^L] - d_L\,[L] && (35)\\
\frac{d[P]}{dt} &= g_P\,([r_S^P]\,[r_T^P] + [r_M^P] - [r_S^P]\,[r_T^P]\,[r_M^P]) - d_P\,[P] && (36)\\
\frac{d[FR]}{dt} &= g_{FR}\,[r_T^{FR}] - d_{FR}\,[FR] && (37)
\end{aligned}\right.$$

References

[1] Greenhill, E., Rocco, A., Vibert, L., Nikaido, M., and Kelsh, R., "An Iterative Genetic and Dynamical Modelling Approach Identifies Novel Features of the Gene Regulatory Network Underlying Melanocyte Development," *PLoS Genetics*, vol. 7, no. 9, p. 18, 2011.

[2] Schwanhäusser, B., Busse, D., Li, N., Dittmar, G., Schuchhardt, J., Wolf, J., *et al.*, "Global quantification of mammalian gene expression control.," *Nature*, vol. 473, no. 7347, pp. 337–342, 2011.

[3] Mertin, S., McDowall, S., and Harley, V., "The DNA-binding specificity of SOX9 and other SOX proteins," *Nucleic Acids Research*, vol. 27, no. 5, pp. 1359–1364, 1999.

[4] Belle, A., Tanay, A., Bitincka, L., Shamir, R., and O'Shea, E., "Quantification of protein half-lives in the budding yeast proteome," *Proceedings of the National Academy of Sciences*, vol. 103, no. 35, pp. 13004–13009, Aug. 2006.

[5] Eden, E., Geva-Zatorsky, N., Issaeva, I., Cohen, A., Dekel, E., Danon, T., *et al.*, "Proteome half-life dynamics in living human cells.," *Science (New York, N.Y.)*, vol. 331, no. 6018, pp. 764–8, Mar. 2011.

[6] Kuhar, M., "Measuring levels of proteins by various technologies: can we learn more by measuring turnover?," *Biochemical pharmacology*, vol. 79, no. 5, pp. 665–8, Mar. 2010.

[7] van de Wetering, M., Oosterwegel, M., van Norren, K., and Clevers, H., "Sox-4, an Sry-like HMG box protein, is a transcriptional activator in lymphocytes.," *The EMBO journal*, vol. 12, no. 10, pp. 3847–54, Oct. 1993.

[8] Connor, F., Cary, P., Read, C., Preston, N., Driscoll, P., Denny, P., *et al.*, "DNA binding and bending properties of the post-meiotically expressed Sry-related protein Sox-5.," *Nucleic acids research*, vol. 22, no. 16, pp. 3339–3346, 1994.

[9] Pogenberg, V., Ögmundsdóttir, M., Bergsteinsdóttir, K., Schepsky, A., Phung, B., Deineko, V., *et al.*, "Restricted leucine zipper dimerization and specificity of DNA recognition of the melanocyte master regulator MITF," *Genes and Development*, vol. 26, no. 23, pp. 2647–2658, 2012.
