# Supplementary material for: A systems biology approach uncovers the core gene regulatory network governing iridophore fate choice from the neural crest
Source: PLoS Genet. 2018 Oct 4;14(10):e1007402. doi: 10.1371/journal.pgen.1007402 (PMC6191144; doi:10.1371/journal.pgen.1007402)
Supplement: S2 Text — (PDF) [file pgen.1007402.s011.pdf]

S2 Text. Monte Carlo scoring functions

We scored the performance for randomly drawn parameters and applied principal component analysis (PCA) to visualise the results using MatLab software. The scoring process is as follows:

1. We defined the variable parameter data set of the problem as all positive initial values, maximum production rates (g), protein degradation rates (d), and dissociation constants for transcription factors binding (K).
2. By random uniform logarithmic draw, we let all data vary in the range between a multiple of 1/3.5 and 3.5 that is exp(±2.5/2).
3. We scored the overall performance (Sc_{total}) of each model according to the following formula:

$$Sc_{total} = Sc_{WT}\, Sc_{sox10}\, Sc_{tfec}\, Sc_{ltk}$$

Where Sc_{WT} is the score in the WT context, in which Mitfa [M] concentration is expected to first rise and the decline, Sox10 [S], Tfec [T] and Ltk [L] concentrations are to be maintained at a positive level in the steady state, and Pnp4a [P] is to reach a relatively high concentration at steady state, without generating oscillations.

$$Sc_{WT} = (risetofall[M])(tailexists[S])(maintaintail[T])$$
$$(tailexists[L])(noosctail[P])(taillarge[P])$$

Sc_{sox10}, Sc_{tfec} and Sc_{ltk} refer to the scores of a particular model when simulating *sox10*, *tfec* and *ltk* loss of function, respectively. The formulae for calculating these scores are the following:

$$Sc_{sox10} = (undetectable[M])(undetectable[P])(tailexists[T])$$
$$(tailexists[L])(tailexists[S])$$

$$Sc_{tfec} = (risengone[M])(risengone[T])(undetectable[L])(risengone[S])$$

$$Sc_{ltk} = (risengone[T])(risengone[L])(risengone[P])$$

The individual features of each gene, which are used to calculate the above scores are estimated according to the following code:

```
function s=tailexists(tail)
s=tail>0.1;
end;

function s=undetectable(maxv)
s=maxv<0.1;
end;

function s=risetofall(maxv,tail)
s=(maxv>0.05)*4*(2-exp(1-maxv/(tail+1e-
6)))^2;
end;

function s=noosctail(last,tail)
s=(0+exp(-abs(tail-last)));
end;

function s=maintaintail(maxv,tail)
s=(maxv>0.1)*2*(2-exp(1-tail/maxv));
end;

function s=taillarge(tail)
s=tail>0.3;
end;

function s=risengone(maxv,tail)
s=(tail<0.1)*(2-exp(0.1-maxv));
end;
```

In the above, the 'tailexists' and 'undetectable' scores are hard constraints, i.e. if they fail a threshold (0.1 nM) the returned score is zero, resulting in $Sc_{total} = 0$.
